# Interpretation of cell mechanical experiments in microfluidic systems depend on the choice of cellular shape descriptors

**DOI:** 10.1063/5.0084673

**Published:** 2022-04-28

**Authors:** Bob Fregin, Doreen Biedenweg, Oliver Otto

**Affiliations:** 1 Zentrum für Innovationskompetenz-Humorale Immunreaktionen bei kardiovaskulären Erkrankungen, Universität Greifswald, Friedrich-Ludwig-Jahnstrasse 15a, 17489 Greifswald, Germany; 2 Institut für Physik, Universität Greifswald, Felix-Hausdorff-Strasse 6, 17489 Greifswald, Germany; 3 Deutsches Zentrum für Herz-Kreislauf-Forschung e.V., Standort Greifswald, Universitätsmedizin Greifswald, Fleischmannstrasse 42, 17489 Greifswald, Germany; 4 Universitätsmedizin Greifswald, Fleischmannstrasse 8, 17489 Greifswald, Germany

## Abstract

The capability to parameterize shapes is of essential importance in biomechanics to identify cells, to track their motion, and to quantify deformation. While various shape descriptors have already been investigated to study the morphology and migration of adherent cells, little is known of how the mathematical definition of a contour impacts the outcome of rheological experiments on cells in suspension. In microfluidic systems, hydrodynamic stress distributions induce time-dependent cell deformation that needs to be quantified to determine viscoelastic properties. Here, we compared nine different shape descriptors to characterize the deformation of suspended cells in an extensional as well as shear flow using dynamic real-time deformability cytometry. While stress relaxation depends on the amplitude and duration of stress, our results demonstrate that steady-state deformation can be predicted from single cell traces even for translocation times shorter than their characteristic time. Implementing an analytical simulation, performing experiments, and testing various data analysis strategies, we compared single cell and ensemble studies to address the question of computational costs vs experimental accuracy. Results indicate that high-throughput viscoelastic measurements of cells in suspension can be performed on an ensemble scale as long as the characteristic time matches the dimensions of the microfluidic system. Finally, we introduced a score to evaluate the shape descriptor-dependent effect size for cell deformation after cytoskeletal modifications. We provide evidence that single cell analysis in an extensional flow provides the highest sensitivity independent of shape parametrization, while inverse Haralick's circularity is mostly applicable to study cells in shear flow.

## INTRODUCTION

Mechanical properties of biological systems, e.g., cells and tissues, provide a direct link to their function and can be seen as a biomarker^1–5^ in cell development, cell state determination, and disease screening.^2,4–7^ As rheological tests are based on the application of external stresses while monitoring strain, a correct definition of cell deformation is of key importance.^5,8,9^ Various shape descriptors enable parametric analyses based on geometrical features like size, perimeter, circularity, and membrane curvature. Their potential has already been demonstrated in establishing a link between the cell function and shape for the migration of adherent cells and to investigate the potential of morphological features for a quantitative analysis of cell microscopy images in general.^8,9^ In contrast, a systematic analysis of shape descriptors for cells in suspension is currently elusive. The lack of standardization makes it difficult to compare viscoelastic properties quantified by different microfluidic methods.

Earlier work investigated red blood cell (RBC) shapes and dynamics in microchannels of different size and at different flow rates.^10,11^ Experiments have been compared to simulations assuming the RBC membrane as a triangulated network of springs where fluid flow has been modeled by dissipative particle dynamics^10^ or an immersed boundary algorithm was used, solving the evolution of the moving deformable membrane of RBCs overlayed on a static Eulerian domain.^11^ While simulations predict well-defined RBC states at given hydrodynamic conditions, experimental data show a broad distribution of shapes.^10^

RBCs deformed into the shape of parachutes have been analyzed for their shear modulus, determined from a confinement parameter, the Taylor deformation, of a cell-enclosing ellipse.^11^ The shear modulus of RBCs was shown to increase with aging. The role of deformation and shape of RBCs has also been investigated for patients suffering from diabetes with normal and high levels of cholesterol where Babu reports that deformation is reduced in individuals with high cholesterol.^12^ Membrane bending of RBCs is also highly relevant for the investigation of malaria disease pathogenesis. Koch *et al.* demonstrated that the malaria parasite locally reduces the bending modulus to facilitate invasion.^13^ RBCs under pathological conditions have been compared within different microchannel geometries based on the shape parameters like Taylor deformation and axes ratio of a bounding box, where hyperbolic-shaped constrictions yielded the highest sensitivity to small changes in deformability due to the strong extensional flow.^14^

High-throughput assays to quantify the mechanical properties of suspended cells have long been limited to RBCs and other soft particles like hydrogel beads since hydrodynamic shear and normal stress were insufficient to induce a finite deformation in nucleated cells. The advent of technologies like deformability cytometry^3^ and real-time deformability cytometry^15–17^ expanded the applicability of microfluidic systems to stiffer objects like white blood cells by exploiting the possibilities of inertia-driven flows or by increasing the viscosity of the measurement buffer. These approaches enabled mechanical phenotyping of nearly all cell types and has been used in basic and translational research, e.g., to identify and characterize hematopoietic and mesenchymal stem cells,^15,18–20^ to monitor the onset and progression of diseases^21^ and as a quality control in translational medicine.^22^ While these studies and others contributed a lot to our understanding of life sciences from a biophysical perspective, a systematic description of how shape quantification impacts on deformation analysis is still lacking.

In our work, we compared nine different shape descriptors to characterize the deformation of suspended cells in an extensional as well as shear flow, which originates from microfluidic geometries found in almost any lab-on-chip design. A detailed understanding of how hydrodynamics and shape parametrization impacts on stress distribution and strain quantification, respectively, is of utmost importance for the interpretation of data from any rheological assay that investigates cells in motion. We previously had shed light on the role of hydrodynamic stress for the shape evolution of cells^17^ and introduced a framework for statistical data evaluation.^23^ Now we expand our analyses toward the comparison of nine parameters describing cell shapes that are being used in stress relaxation or creep-compliance experiments.^3,5,15,24–27^ Those descriptors include cell area, front radius, axes ratio, Taylor deformation of a bounding box, principal axes ratio, rescaled circularity, inverse Haralick's circularity, circular variance, and inertia ratio.

Within the present study, we first monitored the evolution of cell shape in response to an extensional as well as shear flow. While stress relaxation dynamics differ for all shape descriptors, our results demonstrate that steady-state deformation can be predicted from single cell traces even for translocation times shorter than its characteristic time. This is an essential requirement to extract material properties from stress–strain experiments. Second, we compared single cell vs ensemble studies to address the question of computational effort vs experimental accuracy. Our data indicate the necessity of analyzing single cell traces for short or unknown relaxation times, i.e., for cells with unknown material properties. Finally, we investigated how cytoskeletal modifications impact on cell deformation represented by different shape descriptors. As a main contributor to mechanical stability, actin polymerization was inhibited by cytochalasin D (CytoD), and the size of this effect has been calculated as a function of flow geometry, i.e., extensional vs shear flow, of analysis strategy, i.e., single cell vs ensemble and of the shape descriptors investigated within this work. Single cell analysis in response to an extensional flow provides highest sensitivity independent of how deformation is quantified while inverse Haralick's circularity provides highest score in effect size for cells in shear flow. This experiment is of fundamental importance in mechanobiology to link alterations on a molecular scale to a cellular phenotype.

## MATERIALS AND METHODS

### Experimental setup

A microfluidic system described in Refs. 15 and 17 is used. Briefly, a narrow microfluidic channel of 30 × 30 *μ*m^2^ cross section and 300 *μ*m length fabricated facilitating soft-lithography is assembled on a standard inverted microscope as part of the AcCellerator system (Zellmechanik Dresden). While cells in suspension pass the constriction, they are illuminated by a high-power pulsed LED (L1, Zellmechanik Dresden) and imaged using a high-speed CMOS camera (MC1362, Mikrotron) as well as a 40x objective (A-Plan, NA 0.65, Zeiss) leading to a resolution of 0.34 *μ*m per pixel. Sample suspension as well as a sheath flow for hydrodynamic focusing into the channel are connected via tubing to and controlled by a two-channel syringe pump (Nemesys, Cetoni). An outlet is connected to a waste reservoir.

### Cell culture

All experiments have been performed using the model system HL60, a myeloid precursor suspension cell line (courtesy of Dan and Ada Olins). Cells are cultured in an RPMI-1640 medium (BioWest) with 10% FCS (Gibco), 1% penicillin/streptomycin (BioWest), and 2 mM L-Glutamin (BioWest) in a standard incubator at 37 °C, 5% CO_2_, and 95% air. Cells are split approximately every 48 h by centrifugation for 5 min at 200 rcf (Allegra X-15R, Beckman Coulter). The supernatant is discarded and the cells are re-suspended in medium. Concentration is adjusted to approximately 1.5 × 10^5^ cells/ml. Measurements were performed during the log phase, approximately 36 h after splitting. Viability of cells has been assessed to ∼95% using Trypan blue. Cells responded negatively to a test for Mycoplasma infection (MycoSPY®, Biontex).

### Experimental assay

Dynamic real-time deformability cytometry (dRT-DC) assays are carried out as reported earlier.^17^ Briefly, cells are centrifuged for 5 min at 200 rcf (Allegra X-15R, Beckman Coulter) and re-suspended in phosphate-buffered saline (PBS−/−, without Ca^2+^/Mg^2+^, BioWest) complemented with 1% (w/v) methylcellulose (Sigma-Aldrich) as the measurement buffer. First, the microfluidic chip is filled with the buffer in sheath flow. Second, 100 *μ*l of the sample solution are drawn into the tubing and connected to the sample inlet of the chip. The channel is flushed with the two flows at a flow rate of 100 nl s^−1^ each. After dosing 10 *μ*l with the two 1 ml syringes (1 ml Luer-Lock syringe, BD), the target flow rate of 8 nl s^−1^ (2 nl s^−1^ for sample and 6 nl s^−1^ for sheath flow) is set. Dynamic RT-DC acquisition is started after 12 min of equilibration time. Proper alignment of cells in the center of the channel is guaranteed by hydrodynamic focusing.

### Inhibition of actin polymerization

Cytochalasin D (Sigma-Aldrich) dissolved in dimethylsulfoxide (DMSO) is added at a concentration of 1 *μ*M to HL60 cells (approximately 1 × 10^6^ cells per milliliter).^28^ Cells are incubated for 10 min at 37 °C, centrifuged for 5 min at 200 rcf (Allegra X-15R, Beckman Coulter), and re-suspended in PBS−/− complemented with 1% (w/v) methylcellulose (Sigma-Aldrich) as the measurement buffer. Corresponding vehicle is treated with 0.25% (v/v) DMSO while the control condition is untreated.

### Cell shape analysis

**FIG. 1.**
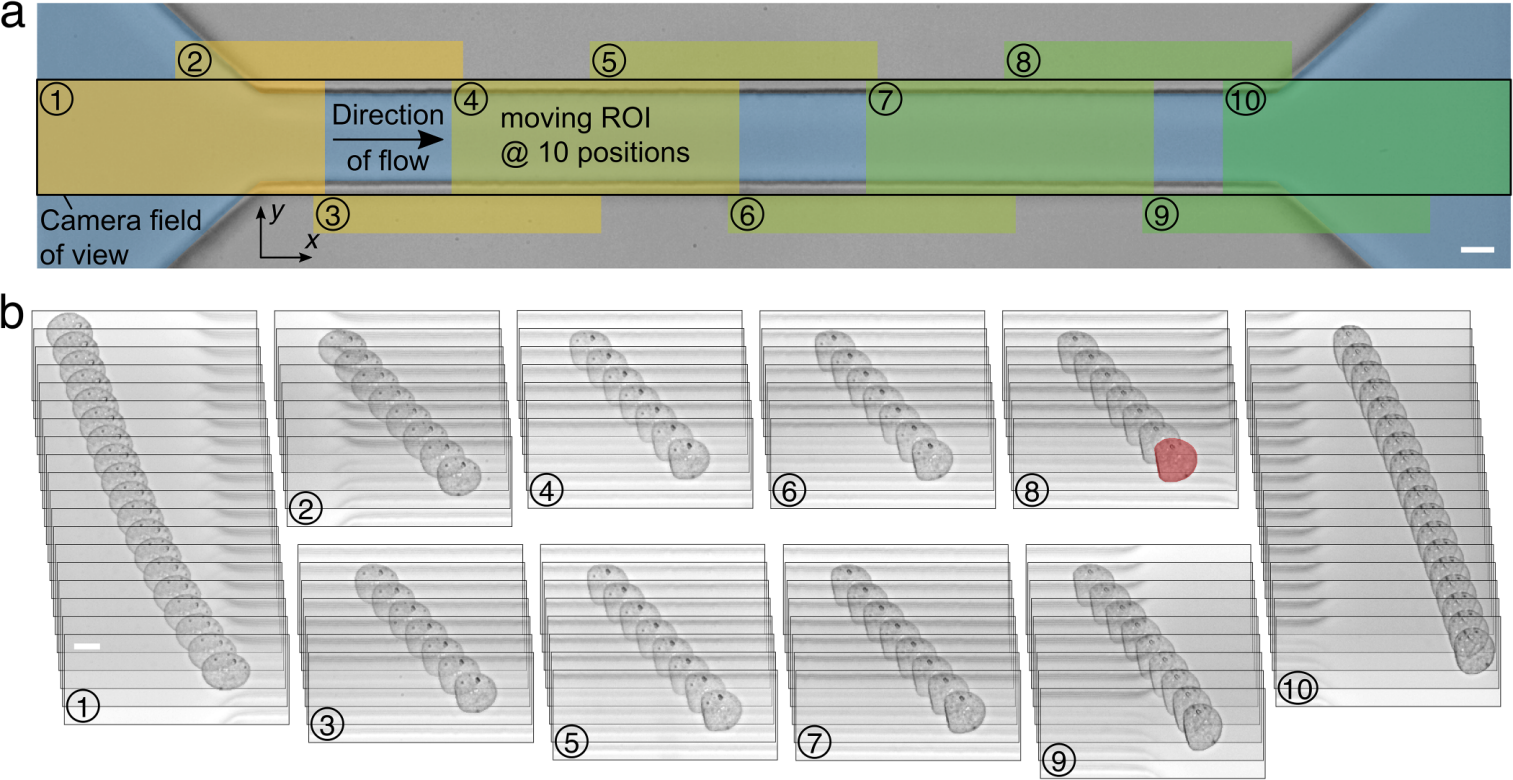
Dynamic tracking of single cells in a microfluidic channel. (a) Image of a 30 × 30 *μ*m^2^ microfluidic channel of 300 *μ*m length. Overlaid sketch demonstrates the principle of dynamic cell tracking. Camera field of view is indicated by a black rectangle covering the complete length of the channel as well as the inlet and outlet regions. A moving cell is tracked by a moving region-of-interest (ROI, 250 × 100 pixels) shifted stepwise in the direction of flow depending on the cell position inside the channel (ROI positions 1–10 are highlighted). (b) Image sequence of a tracked HL60 cell captured over time consisting of 98 consecutive frames. The trace was captured at the ten different ROI positions indicated in (a). The cell shape at the end of the channel is highlighted in red and scale bars are 10 *μ*m. Flow rate was set to 8 nl s^−1^.

Cell deformation analysis is carried out in a custom software for dynamic cell tracking^17^ adopted from Ref. 15. The camera acquires images at 3000 frames per second within a field of view of 1280 × 100 pixels, equivalent to 435 × 34 *μ*m^2^, covering the complete channel length including inlet and outlet regions [Fig. 1(a)]. To reduce the processing bandwidth, the tracking algorithm analyzes a smaller region-of-interest (ROI) within the full field of view by cropping of 250 × 100 pixels around the moving cell. This moving ROI encapsulating the tracked cell starts at the left boundary and is shifted stepwise by 120 pixels to the right (with increasing *x*) if the cell's center of mass (COM) passes a threshold in the cropped image at approximately 70% of ROI width. Image acquisition and processing is implemented in LabView (LabView 2018, National Instruments) utilizing functions of the computer vision library OpenCV (https://opencv.org/) compiled into dynamic link libraries. After background subtraction, a binary image is generated by comparing the 8-bit image to a threshold of gray scale value 6. The binarized image enables first a border-following algorithm^29^ and second a convex hull algorithm^30^ to find contour coordinates, which are saved and used to calculate a rescaled circularity $c^{*}$ as a deformation measure,

$$c^{*}=1-c=1-\frac{2\sqrt{\pi A}}{P},$$ (1)

where *c*, *A*, and *P* represent the circularity, the projected cell area, and the perimeter, respectively. Image acquisition, processing, and cell shape analysis is done in real-time during the experiment.

Before entering the channel, cells move slowly and, consequently, shear forces are small resulting into undeformed cell shapes close to a circle ( $c^{*}\approx 0$), whereas cells are stretched into an ellipsoidal shape at the inlet due to an acceleration in the direction of flow [Fig. 1(b)]. Inside the channel, cells follow the Poiseuille flow profile and mimic a bullet-shaped steady-state at the channel end.

For each cell and at each position within the field of view, the bright-field images, contour coordinates, as well as area and circularity are stored during the experiment for later analysis. In a post-processing step, cell contour coordinates are used to calculate the time traces of the remaining shape descriptors.

### Cell shape descriptors

Deformation of a cell is quantified by multiple shape descriptors. In the following, we present the definition of the cell area, the normalized front radius, the axes ratio, the Taylor deformation, the principal axes ratio, the rescaled circularity, inverse Haralick's circularity, circular variance, and the inertia ratio.^9,31,32^

All calculations are based on a convex hull cell contour described by a closed polygon $\left(x_{n},y_{n}\right)$ with *N* points where n=0,1,…,N−1 and $\left(x_{N},y_{N})=(x_{0},y_{0}\right)$. The cell is aligned in the direction of flow, which we assign the *x* direction [Fig. 1(a)].

During image acquisition, cell contour is initially stored in Cartesian coordinates. For the shape descriptors’ principal axes ratio, inverse Haralick's circularity, and circular variance, the contour is interpreted as an angle-dependent radius function r(φ) and needs to be transformed into polar coordinates. To ensure equidistant sampling in φ space, the contour is piecewise linearly interpolated to 500 points in total over 2π in Cartesian coordinates $\left(x_{\mathrm{interp},k},y_{\mathrm{interp},k}\right)$. Interpolation is necessary to ensure that calculation of shape descriptors is insensitive against the number of contour points. Here, a 500 point interpolation yields an error below 0.1% (Fig. S1 in the supplementary material). Interpolated points are transformed into polar coordinates $r_{\mathrm{interp},k}(\varphi_{\mathrm{interp},k})$,

$$\begin{array}{rl} & r_{\mathrm{interp},k}=\sqrt{{\left(x_{\mathrm{interp},k}-x_{\mathrm{COM}}\right)}^{2}+{\left(y_{\mathrm{interp},k}-y_{\mathrm{COM}}\right)}^{2}},\\ & \varphi_{\mathrm{interp},k}=\arctan2(y_{\mathrm{interp},k}-y_{\mathrm{COM}},x_{\mathrm{interp},k}-x_{\mathrm{COM}}), \end{array}$$ (2)

where $\left(x_{\mathrm{COM}},y_{\mathrm{COM}}\right)$ represents the coordinates of center of mass with

$$\begin{array}{r} x_{\mathrm{COM}}=\frac{1}{6A}\underset{n}{\sum }(x_{n}+x_{n+1})(x_{n}y_{n+1}-x_{n+1}y_{n}),\\ y_{\mathrm{COM}}=\frac{1}{6A}\underset{n}{\sum }(y_{n}+y_{n+1})(x_{n}y_{n+1}-x_{n+1}y_{n}),\\ \mathrm{with}\;\mathrm{Area}\;A=\frac{1}{2}\left|\underset{n}{\sum }(x_{n}y_{n+1}-x_{n+1}y_{n})\right|.\; \end{array}$$ (3)

**Area:** Cell area *A* is defined as the cell area enclosed by its convex hull contour [Eq. (3)].

**FIG. 2.**
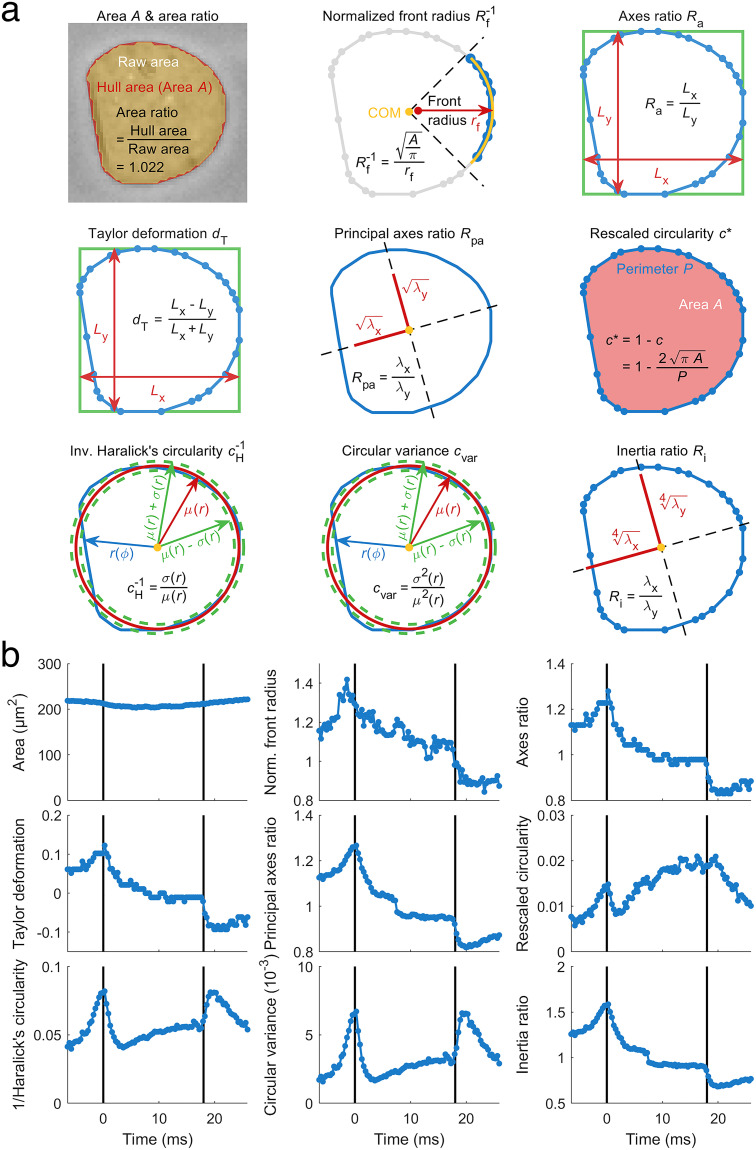
Shape descriptors reveal varying cell response. (a) Nine shape descriptors are introduced and applied to the steady-state shape of a HL60 cell [highlighted cell shape at the channel end in Fig. 1(b)]. Cell shape is represented by blue contour points and connecting line segments. (b) Time-dependent traces of deformation represented by the different shape descriptors introduced in (a) and applied to the deformed shapes of a representative HL60 cell passing a 30 × 30 *μ*m^2^ channel of 300 *μ*m length at a flow rate of 8 nl s^−1^.

**Normalized front radius:** The normalized front radius $R_{f}^{-1}$ represents the curvature of the cell's front part in the direction of flow [Fig. 1(a)]. For calculation, contour points under an angle of 90° of the leading part relative to the center of mass are considered [ $-45^{{}^{\circ}}\leq \varphi_{n}\leq 45^{{}^{\circ}}$, Fig. 2(a)]. A least squares approximation enables calculating the center $\left(x_{c},y_{c}\right)$ of the corresponding circle, from which the front radius $r_{f}$ can be derived,

$$\begin{array}{r} r_{n}(x_{c},y_{c})=\sqrt{{\left(x_{n}-x_{c}\right)}^{2}+{\left(y_{n}-y_{c}\right)}^{2}},\\ \underset{x_{c},y_{c}}{\min}\underset{n}{\sum }{\left(r_{n}(x_{c},y_{c})-\bar{r_{n}}(x_{c},y_{c})\right)}^{2},\\ r_{f}=\bar{r_{n}}(x_{c},y_{c}).\;\;\;\;\; \end{array}$$ (4)

If the minimization algorithm fails to find the global minimum, which is examined from a comparison of the returned $r_{f}$ with an upper threshold, set to $10\sqrt{A/\pi }$, the front radius at the current cell position needs to be excluded.

For a size-independent representation, front radius is normalized to the equivalent radius of a cell with circular shape and of same size ( $\sqrt{A/\pi }$). The front radius has a local minimum at the inlet in contrast to all other shape descriptors but Haralick's circularity. For comparison, we analyze the inverse normalized front radius here, referred to as the normalized front radius $R_{f}^{-1}$,

$$R_{f}^{-1}=\frac{\sqrt{\frac{A}{\pi }}}{r_{f}}.$$ (5)

**Axes ratio:** The axes ratio $R_{a}$ is defined by the side lengths of the bounding box fully enclosing the cell [Fig. 2(a)] and is calculated from length $L_{x}$ along the axis in the direction of flow divided by length $L_{y}$ along the perpendicular axis,

$$R_{a}=\frac{L_{x}}{L_{y}}=\frac{\max(x_{n})-\min(x_{n})}{\max(y_{n})-\min(y_{n})}.$$ (6)

**Taylor deformation:** Taylor deformation $d_{T}$ is defined as the difference of the two axis lengths of the bounding box divided by their sum [Fig. 2(a)],

$$d_{T}=\frac{L_{x}-L_{y}}{L_{x}+L_{y}}.$$ (7)

**Principal axes ratio:** The principal axes ratio $R_{\mathrm{pa}}$ is defined by the two principal axes lengths of a cell contour and is calculated from a covariance matrix *C* based on interpolated cell contour points relative to its center of mass [Fig. S1 in the supplementary material, cf. Eq. (2)],

$$\begin{array}{r} \vec{x}=x_{\mathrm{interp},k}={\left[\begin{array}{cccc} x_{0} & x_{1} & \ldots & x_{499} \end{array}\right]}^{T},\;\;\;\\ \vec{y}=y_{\mathrm{interp},k}={\left[\begin{array}{cccc} y_{0} & y_{1} & \ldots & y_{499} \end{array}\right]}^{T},\;\;\;\\ C=\frac{1}{500}{\left[\begin{array}{cc} \vec{x}-x_{\mathrm{COM}} & \vec{y}-y_{\mathrm{COM}} \end{array}\right]}^{T}\cdot \left[\begin{array}{cc} \vec{x}-x_{\mathrm{COM}} & \vec{y}-y_{\mathrm{COM}} \end{array}\right]\\ =\left[\begin{array}{cc} C_{xx} & C_{xy}\\ C_{yx} & C_{yy} \end{array}\right].\;\;\;\;\;\;\; \end{array}$$ \vskip-11pt (8)

$R_{\mathrm{pa}}$ is defined as the ratio of both eigenvalues $\lambda_{1,2}$ of *C* [Fig. 2(a), square roots are depicted since eigenvalues have the dimension length to the power of two]. To ensure correct assignment, corresponding eigenvectors are filtered for their orientation.

Here, the principal axes ratio is calculated as the eigenvalue corresponding to the eigenvector dominated by the axis in direction of flow ( x) divided by its perpendicular counterpart ( y),

$$\begin{array}{r} C{\vec{v}}_{i}=\lambda_{i}{\vec{v}}_{i},\;\;{\vec{v}}_{i}={\left[\begin{array}{cc} v_{i,x} & v_{i,y} \end{array}\right]}^{T},\;\;i=1,2,\\ R_{\mathrm{pa}}=\{\begin{array}{cc} \frac{\lambda_{1}}{\lambda_{2}}, & |v_{1,x}|>|v_{2,x}|,\\ \frac{\lambda_{2}}{\lambda_{1}}, & \mathrm{else}.\; \end{array}\;\;\; \end{array}$$ (9)

**Rescaled circularity:** The rescaled circularity $c^{*}$ is calculated from the cell circularity *c* according to Eq. (1). For a completely circular cell, the rescaled circularity equals zero.

**Inverse Haralick's circularity:** Haralick's circularity $c_{H}$ is based on the radial distribution of all cell contour points and is calculated from the interpolated radii mean ${\bar{r}}_{\mathrm{interp}}$ divided by their standard deviation^33^ [cf. Eq. (2)]. In contrast to most shape descriptors, $c_{H}$ possesses a local minimum at the channel inlet and we, therefore, use the inverse Haralick's circularity $c_{H}^{-1}$ in this work, featuring improved numerical stability since the standard deviation $\sigma (r_{\mathrm{interp}})$ is shifted from the denominator to the nominator [Fig. 2(b)],

$$\begin{array}{r} c_{H}^{-1}=\frac{\sigma (r_{\mathrm{interp}})}{\mu (r_{\mathrm{interp}})},\;\;\;\;\;\;\\ \sigma (r_{\mathrm{interp}})=\sqrt{\frac{1}{499}\underset{k}{\sum }{\left(r_{\mathrm{interp},k}-{\bar{r}}_{\mathrm{interp}}\right)}^{2}},\\ \mu (r_{\mathrm{interp}})={\bar{r}}_{\mathrm{interp}}=\frac{1}{500}\underset{k}{\sum }r_{\mathrm{interp},k}.\;\; \end{array}$$ (10)

**Circular variance:** The circular variance $c_{\mathrm{var}}$ relates the radial variance to the squared radii mean [Eq. (2)],

$$\begin{array}{r} c_{\mathrm{var}}=\frac{\mathrm{Var}(r_{\mathrm{interp}})}{\mu^{2}(r_{\mathrm{interp}})},\;\;\\ \mathrm{Var}(r_{\mathrm{interp}})=\sigma^{2}(r_{\mathrm{interp}}). \end{array}$$ (11)

Circular variance is the square of inverse Haralick's circularity.

**Inertia ratio:** The inertia ratio $R_{i}$ relates the area moment of inertia along the two axes of minimal and maximal moment of area [Fig. 2(a), depicted are the fourth roots since the moment of inertia has the dimension length to the power of four]. First, the area moments of inertia $I_{xx}$, $I_{yy}$, and $I_{xy}$, related to the *x* axis, *y* axis, and their diagonal, respectively, are calculated for contour points relative to their center of mass $\left({\tilde{x}}_{n},\;{\tilde{y}}_{n})=(x_{n}-x_{\mathrm{COM}},\;\;y_{n}-y_{\mathrm{COM}}\right)$,

$$\begin{array}{r} I_{xx}=\frac{1}{12}\underset{n}{\sum }({\tilde{x}}_{n}{\tilde{y}}_{n+1}-{\tilde{x}}_{n+1}{\tilde{y}}_{n})({\tilde{y}}_{n}^{2}+{\tilde{y}}_{n}{\tilde{y}}_{n+1}+{\tilde{y}}_{n+1}^{2}),\\ I_{yy}=\frac{1}{12}\underset{n}{\sum }({\tilde{x}}_{n}{\tilde{y}}_{n+1}-{\tilde{x}}_{n+1}{\tilde{y}}_{n})({\tilde{x}}_{n}^{2}+{\tilde{x}}_{n}{\tilde{x}}_{n+1}+{\tilde{x}}_{n+1}^{2}),\\ I_{xy}=\frac{1}{24}\underset{n}{\sum }({\tilde{x}}_{n}{\tilde{y}}_{n+1}-{\tilde{x}}_{n+1}{\tilde{y}}_{n})({\tilde{x}}_{n}{\tilde{y}}_{n+1}+2{\tilde{x}}_{n}{\tilde{y}}_{n}\\ +\;2{\tilde{x}}_{n+1}{\tilde{y}}_{n+1}+\;{\tilde{x}}_{n+1}{\tilde{y}}_{n}). \end{array}$$ \vskip-10pt (12)

The eigenvalues and eigenvectors of the area moment of inertia tensor *T* are determined (describing minimal and maximal moment of area and corresponding axes).

For correct assignment of extremal axes of inertia ratio, corresponding eigenvectors are filtered for orientation with respect to the direction of flow. $R_{i}$ is then calculated as the inertia dominated by axis in the direction of flow ( x) divided by its perpendicular counterpart ( y),

$$\begin{array}{r} T=\left[\begin{array}{cc} I_{xx} & -I_{xy}\\ -I_{xy} & I_{yy} \end{array}\right],\;\;\;\;\\ T{\vec{v}}_{i}=\lambda_{i}{\vec{v}}_{i},\;{\vec{v}}_{i}={\left[\begin{array}{cc} v_{i,x} & v_{i,y} \end{array}\right]}^{T},\;\;i=1,2,\\ R_{i}=\{\begin{array}{cc} \frac{\lambda_{2}}{\lambda_{1}}, & |v_{1,x}|>|v_{2,x}|,\\ \frac{\lambda_{1}}{\lambda_{2}}, & \mathrm{else}.\; \end{array}\; \end{array}$$ \vskip-10pt (13)

### Data filtering strategy

The tracking algorithm derives a convex hull contour of the cell from every raw image. Goodness of the contour fit is determined by comparing the convex hull area to the raw cell area [Fig. 2(a), first panel]. A cell trace is considered to be valid if at least 80% of all images inside the channel possess a convex hull area that is not more than 5% bigger than the raw cell area. If a cell trace does not cover the entire length of the channel, it is excluded from data analysis.

Cells are filtered for projected cell areas within [75, 300] *μ*m^2^ in order to exclude debris as well as cell clusters. Since the stress on cell surface depends on the cell velocity, only cells with a mean velocity v±3σ are considered for analysis, with standard deviation σ.

**FIG. 3.**
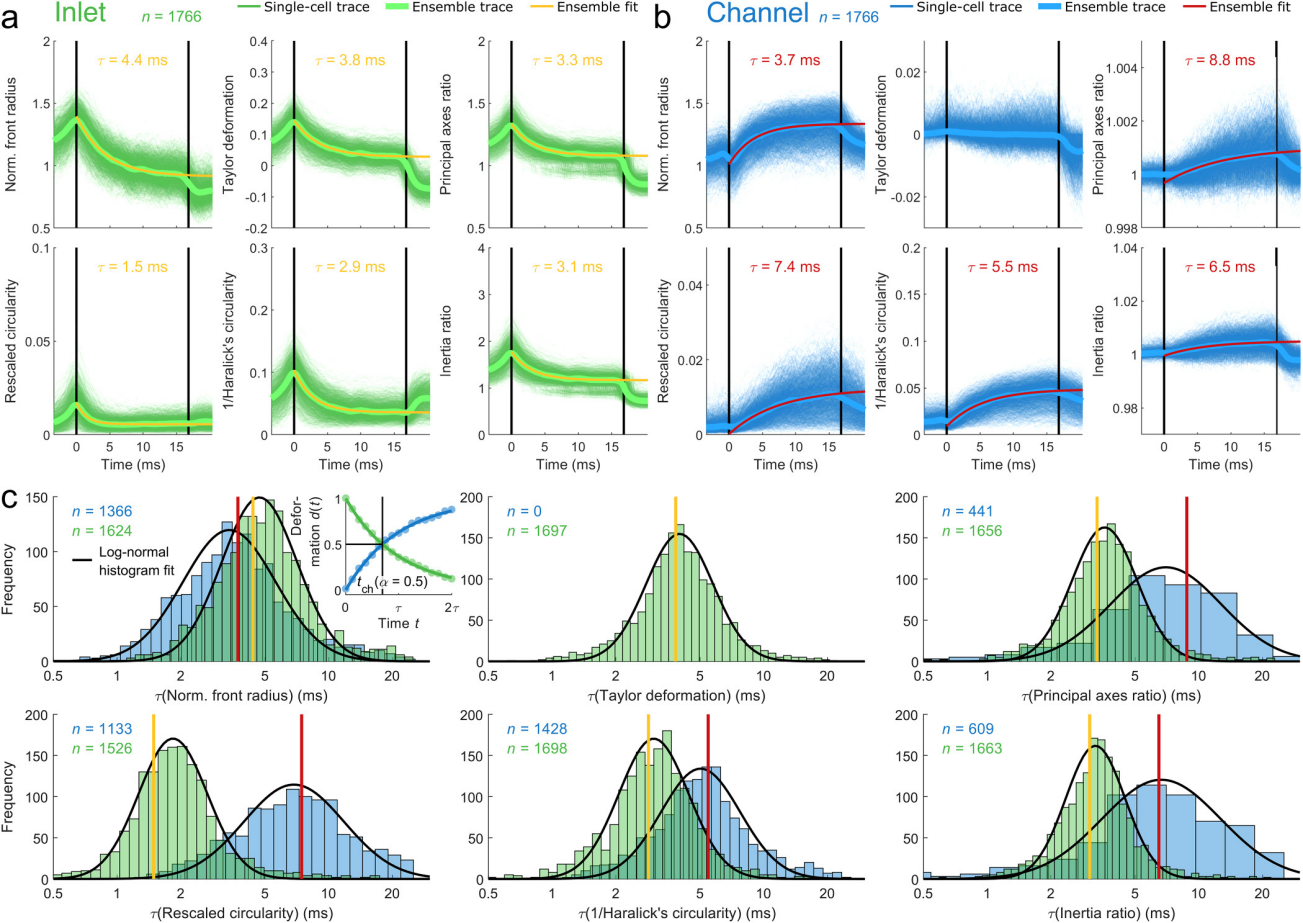
Dynamics of shape descriptors for reconstructed Fourier traces. (a) and (b) Cell shapes are decomposed by Fourier transformation and reconstructed from two subsets of first ten Fourier coefficients (FCs): even FC representing inlet effects [(a), green traces] and odd FC including coefficient zero representing channel effects [(b), blue traces]. Deformation dynamics are shown for 1766 HL60 cells passing a 30 × 30 *μ*m^2^ channel of 300 *μ*m length at a flow rate of 8 nl s^−1^. Ensemble response (median) of all cells was calculated (bold green and bold blue lines) while yellow (a) and red lines (b) represent an exponential fit to median traces. Vertical black lines indicate channel inlet and outlet. (c) Single cell analysis is performed by fitting an exponential function to the data, calculation of characteristic times τ, and filtering the resulting traces for a minimal amplitude fraction of $\alpha_{\min}=0.5$ as well as plotting histograms on logarithmic scale. Distributions are filtered for validity of the fit ( $r^{2}\geq 0.6$). Solid black lines indicate a lognormal fit to the histograms filtering data for ±1.96 σ (95%) with respect to median to reject outliers. Vertical red and yellow lines show a comparison to relaxation times from ensemble fit in (a) and (b). Inset in the top left panel shows an exemplary sketch of deformation d(t) representing an inlet (green dots) and channel (blue dots) trace and corresponding exponential fits (solid lines). Translocation time $t_{\mathrm{ch}}$ is indicated for an amplitude fraction of α=0.5 (vertical black line).

With all our experiments being carried out at a flow rate of 8 nl s^−1^, we find a range of 17.6 ± 1.8 mm s^−1^ for a typical experiment with 1806 cells acquired. After filtering for cell size and velocity, 1766 cells remain in the sample. This corresponds to 2.2% of the cells being excluded [Figs. 3(a) and 3(b)]

### Fourier analysis

The shape dynamics of a cell translocating a microfluidic channel shows a superimposed response to an inlet stress due to its initial acceleration and to a constant stress inside the constriction. Shape mode decomposition utilizing Fourier transformation published earlier enables us to disentangle cellular response to both contributions.^17^ Briefly, cell contours are transformed from Cartesian into polar coordinates with respect to their center of mass.

From the Fourier transformation of this angle-dependent radius function the first ten Fourier coefficients (FCs) are considered. By symmetry arguments only, it was shown that cell response to inlet stress is reflected by the even Fourier components, whereas the odd Fourier components represent cellular response to the stress inside the channel. To discriminate between the two responses, the shapes are reconstructed from even and odd FC subsets (including FC zero) separately. Consequently, every shape of a cell at any position can be decomposed into two contours representing the response to inlet and channel stresses.

### Time-dependent shape descriptor analysis

Deformation traces d(t), with *d* being a time-dependent scalar representing all shape descriptors considered in this work, are the basis for characteristic time analysis, which is needed, e.g., to calculate viscoelastic material properties.

Here, we extract the characteristic time τ by fitting an exponential function to d(t) using a custom script implemented in Matlab R2018b (Mathworks),

$$d_{\mathrm{fit}}(t)=\hat{d}\;\exp\left(-\frac{t}{\tau }\right)+d_{0},$$ (14)

with time *t* and free fitting parameters $\hat{d}$, τ, and $d_{0}$ representing the deformation amplitude of any shape descriptor discussed in this work, the characteristic time, and the steady-state offset deformation, respectively. This approach is used for fitting of traces independently of the respective analysis strategy, i.e., direct ensemble, ensemble analysis of inlet or channel effects, and single cell analysis of both effects.

Inside the channel, the exponential behavior of $d_{\mathrm{fit}}(t)$ can be understood from a Kelvin–Voigt model with characteristic time,

$$\tau_{\mathrm{channel}}=\frac{\eta }{E},$$ (15)

because of the presence of a step stress. Here, η corresponds to the viscosity and *E* to Young's modulus of the cell.

For direct ensemble analysis, we overlay all single traces into a master curve by calculating the median at each point in time inside the channel, using interpolation to synchronize between uneven sampling times of the traces. Fitting the master curve is performed for points in time within a range between 0% and 95% inside the channel to ensure that characteristic times are not affected by the outlet [cf. Fig. 3(b)]. Of note, since the position is defined by the center of mass, a cell can be considered inside the channel while the leading edge already crosses the outlet.

For ensemble analysis of inlet effects, we overlay all single traces into a master curve after Fourier decomposition and reconstruction from even FC. Subsequently, fitting according to Eq. (14) is performed as described above. Channel traces reconstructed from odd FC require the introduction of a lag time corresponding to 10% of the translocation time for fitting. Here, shape descriptors stay constant for a couple of frames before a response to channel stress is observed [Fig. 3(b)].

Single cell analysis is performed for traces reconstructed from even and odd FC analog to the ensemble traces described above. However, the beginning and end point for fitting need to be found for every single trace individually because the responses vary in their behavior over time. For inlet traces, the position of the minimum (if applicable) is determined from a central moving median filter of seven points length, i.e., three points before and after the current time point.

Traces are fitted from channel inlet until minimum position or channel end if no minimum exists. For channel stress traces, the initial lag phase is searched dynamically and traces are fitted from the end of the lag phase until channel outlet.

Quality of fit is assessed from the coefficient of determination $r^{2}$, where we consider all traces with $r^{2}\geq 0.6$ for data analysis.^17^ The coefficient of determination relates the residual sum of squares ( $SS_{\mathrm{res}}$) to the total sum of squares ( $SS_{\mathrm{tot}}$),

$$r^{2}=1-\frac{SS_{\mathrm{res}}}{SS_{\mathrm{tot}}}=\frac{\sum_{i}{\left(d_{i}-d_{\mathrm{fit},i}\right)}^{2}}{\sum_{i}{\left(d_{i}-\bar{d}\right)}^{2}},$$ (16)

where $d_{i}$ is the ith data point, $d_{\mathrm{fit},i}$ is the corresponding fit function value, and $\bar{d}$ is the data mean.

### Statistical analysis

For statistical analysis, a linear mixed-effects model (LMM) is fitted to the dataset consisting of three biological replicates with paired control (control or DMSO vehicle) and treatment (CytoD), as introduced earlier.^23^ First, a linear mixed-effects model is used considering the treatment as the fixed effect and differences between replicates as the random effect. A second model including random effects only represents the null hypothesis. Utilizing a model comparison by maximizing the likelihood function, a p-value can be extracted. This strategy is used for statistical analysis of single cell measurements where datasets consist of thousands of cells. The error of effect sizes is estimated from errors for intercept and treatment from LMM. Propagation of uncertainty is implemented utilizing a first order Taylor expansion.

Statistical analysis for ensemble measurements is performed by a t-test as the number of observations is not sufficient for fitting a linear mixed-effects model. Effect size is represented by normalizing the treatment parameter (CytoD) by the vehicle parameter (control or DMSO). Effect size diminished by unity is expected to be zero for no effect. A one-parametric t-test on this difference is used to test against zero and provides the corresponding p-value.

## RESULTS

### Cellular shape descriptors reveal characteristic dynamics

For investigating how different shape descriptors affect the calculation of mechanical properties, suspended cells are monitored during the translocation of a microfluidic channel [Fig. 1(a)]. We use dynamic real-time deformability cytometry (dRT-DC) to analyze cell shape within a moving region-of-interest (ROI) at ten different positions between channel inlet and outlet^17^ (see the Materials and Methods section). At a typical flow rate of 8 nl s^−1^, the high temporal resolution of our experimental system enables us to observe the dynamic cell response to the hydrodynamic stress distribution for 98 frames [Fig. 1(b)]. The number of acquired frames differs for individual cells and depends strongly on the flow rate.

Derivation of cell material properties from strain requires knowledge of the stress distribution inside our microfluidic system. While stress on a cell can be obtained from an analytical as well as numerical model,^6,34^ multiple shape descriptors are available to quantify strain. Within this work, we investigated nine different shape descriptors as a measure for cell deformation: projected cell area, normalized front radius, Taylor deformation, axes ratio, principal axes ratio, rescaled circularity, inverse Haralick's circularity, circular variance, and inertia ratio [Fig. 2(a), see the Materials and Methods section]. All of these are geometric parameters for characterizing two-dimensional shapes by a scalar, represent different shape features, and possess different properties. Cell rotation inside the channel, e.g., impacts on normalized front radius, Taylor deformation, and axes ratio, whereas the other shape descriptors are rotational invariant, either by definition like area, rescaled circularity, inverse Haralick's circularity, and circular variance, or due to reference to principal axes of cell orientation like principal axes ratio and inertia ratio.

When monitoring the translocation of a single HL60 cell through a microfluidic constriction, the nine shape descriptors reveal characteristic dynamics [Fig. 2(b)]. Whereas area stays constant over time as expected, shape descriptors based on cell axes like axes ratio, Taylor deformation, principal axes ratio, and inertia ratio show a maximum at inlet position and are monotonous decreasing toward the channel outlet. We also observe a decrease in normalized front radius, indicating that curvature of the leading part of a cell decreases, and, hence, radius increases inside the channel. In contrast, rescaled circularity, inverse Haralick's circularity, and circular variance reveal a minimum amplitude between the maximum at channel inlet and steady-state at channel outlet. Here, we define the steady-state as a condition where cell deformation is time-independent.

### Characteristic times depend on cellular shape descriptors

A cell responds to two different stress distributions inside a microfluidic channel: a peak stress at the inlet due to a reduction in cross section and subsequent acceleration, as well as a constant stress inside the channel originating from the Stokes flow.^17^ This superposition results in complex cellular dynamics reflected in the behavior of the geometrical descriptors.

In general, shape descriptors can be discriminated in quantities revealing a minimum in their deformation trace and those with a monotonic behavior [Fig. 2(b)]. Focusing on a deformation measure derived from circularity, we have recently shown that Fourier decomposition of shape modes enables us to disentangle the cell response to the inlet stress from the effect of the channel stress.^17^ Here, we expanded that analytical framework to all nine shape descriptors introduced above [Fig. 2(a)]. Performing dRT-DC, traces from more than 4800 HL60 cells in three independent experimental replicates were acquired, shapes decomposed and reconstructed from even and odd Fourier components individually. These represent cell response to inlet and channel stress, respectively (see the Materials and Methods section).

Shape descriptors reconstructed from only even Fourier coefficients reveal a peak at the channel entrance and a constant amplitude toward the outlet [Fig. 3(a) and Fig. S2(a) in the supplementary material]. In contrast, most shape descriptors reconstructed from only odd Fourier coefficients possess a constant magnitude at channel inlet, which increases until the cell leaves the constriction [Fig. 3(b) and Fig. S2(b) in the supplementary material]. Remarkably, axes ratio and Taylor deformation are both insensitive to channel stress and stay constant around one and zero, respectively. As expected, projected cell area does not change as a function of time and is, therefore, excluded from further analysis [Figs. S2(a) and S2(b) in the supplementary material]. For simplicity, data for axes ratio and circular variance are displayed in the supplementary material only due to their mathematical similarity to Taylor deformation and inverse Haralick's circularity, respectively [Figs. S2(a) and S2(b) in the supplementary material].

We analyzed the traces of all shape descriptors, first, by obtaining an ensemble average and, second, on a single cell level. The ensemble average [Fig. 3(a), bold green line, and Fig. 3(b), bold blue line] has been calculated from the median over all single cell traces [Fig. 3(a), green traces, and Fig. 3(b), blue traces] for each of the 85 interpolated positions within the microfluidic system and separated for inlet and channel effects. Fitting an exponential function [Eq. (14)] to the ensemble trace yields two characteristic times, one for the cell response to inlet stress [ $\tau_{\mathrm{inlet}}$, Fig. 3(a), yellow lines] and one for the cell response to the constant channel stress [ $\tau_{\mathrm{channel}}$, Fig. 3(b), red lines]. For the latter, $\tau_{\mathrm{channel}}$ can be interpreted as the response of a cell [Eq. (15)],

$$\tau_{\mathrm{channel}}=\frac{\eta }{E},$$

to a creeping-flow experiment exploiting a Kelvin–Voigt model.^27,35–37^ With *E* corresponding to the cellular Young's modulus and η representing its viscosity, the characteristic channel time $\tau_{\mathrm{channel}}$ contains the full viscoelastic information of the cell in the presence of a step stress. The transition into the channel, i.e., the deformation trace $d_{\mathrm{fit}}(t)$ in response to the peak stress at the inlet, can also be described by an exponential function. While the time-dependent and non-uniform stress distribution renders the derivation of Young's modulus and viscosity from analytical models challenging, the corresponding characteristic time $\tau_{\mathrm{inlet}}$ can still be understood as a quantity that contains the full viscoelastic information of the cell in response to the peak stress at the channel inlet. Both $\tau_{\mathrm{inlet}}$ and $\tau_{\mathrm{channel}}$ represent two material parameters and are investigated in this work.

Next, we analyzed our data on a single cell level [Figs. 3(a) and 3(b), green and blue traces] by fitting an exponential function [Eq. (14)] to every inlet and channel trace [Fig. 3(c), inset top left panel]. Goodness of the exponential fit is assessed by the coefficient of determination ( $r^{2}$, see the Materials and Methods section). Only fits with $r^{2}\geq 0.6$ are included in our analysis, as we already demonstrated that this threshold provides a good data representation.^17^ Comparison between different conditions further requires that experimental timescales given by the channel translocation time $t_{\mathrm{channel}}$ match the characteristic timescales $\tau_{\mathrm{inlet}}$ and $\tau_{\mathrm{channel}}$ of individual cells.

This is specifically of importance when analyses require cells being in steady-state, which corresponds to five times the characteristic time (equivalent to more than 99% of asymptotic value of an exponential function for t→∞). This implies that a relaxation time of, e.g., 5 ms requires a translocation time of 25 ms.

For investigating the steady-state condition, we introduced an amplitude fraction α as the ratio between the shape descriptor amplitude at the channel outlet $d(t_{\mathrm{channel}})$ and the steady-state amplitude d(t→∞), both corrected for the amplitude at the channel inlet d(t=0), all obtained from the exponential fit,

$$\alpha =\frac{d(t_{\mathrm{channel}})-d(t=0)}{d(t\to \infty )-d(t=0)}.$$ (17)

If the amplitude fraction of an experiment exceeds $\alpha \geq \alpha_{\min}$ (Fig. S3 in the supplementary material), the characteristic time from the fit is considered to be a valid representation of the cellular relaxation time. Utilizing the exponential dependency between the amplitude and time domain, Eq. (17) can be rewritten by means of the relevant time scales [Eqs. (1)–(5) in the supplementary material],

$$\tau \leq \tau_{\mathrm{threshold}}\;,\;\;\tau_{\mathrm{threshold}}=\frac{t_{\mathrm{channel}}}{-\ln(1-\alpha_{\min})}.$$ (18)

Equation (18) enables the definition of an upper threshold $\tau_{\mathrm{threshold}}$ of valid characteristic times for single cells by measuring a median translocation time and imposing a minimal amplitude fraction $\alpha_{\min}$.

In a typical experimental setting with a 300 *μ*m long channel and applying a flow rate of 8 nl s^−1^, we find a mean cell velocity of 17.6 mm s^−1^ for HL60 cells and a channel translocation time of 17.0 ms (Fig. S4 in the supplementary material, top left panel). For $\alpha_{\min}=0.5$, i.e., at least 50% of a given steady-state amplitude is found inside the channel [Fig. 3(c), inset top left panel]; the threshold in characteristic time is given by $\tau_{\mathrm{threshold}}=1.44\cdot t_{\mathrm{channel}}$, which corresponds to the condition $t_{\mathrm{channel}}\geq 0.69\cdot \tau_{\mathrm{inlet}/\mathrm{channel}}$ [Eq. (18)].

In our work, we analyzed n=4845 single cell traces from three biological replicates by Fourier decomposition and subsequent shape reconstruction from odd as well as even coefficients. Applying a minimal amplitude fraction $\alpha_{\min}=0.5$ as a selection criterion for valid exponential fits, the resulting characteristic times are described by a lognormal distribution [Fig. 3(c) and Fig. S4 in the supplementary material, one replicate is presented]. In general, we find $\tau_{\mathrm{channel}}>\tau_{\mathrm{inlet}}$, which can be explained by the short peak stress at the inlet and the longer constant stress in the channel. Interestingly, traces described by the normalized front radius demonstrate an opposite behavior, i.e., $\tau_{\mathrm{channel}}<\tau_{\mathrm{inlet}}$, a result that potentially originates from its sensitivity toward cell contour curvature in the direction of flow. In contrast, traces quantified by axis ratio and Taylor deformation are nearly insensitive toward the constant channel stress and the amplitude of the shape descriptors remains unaltered [Fig. 3(b) and Fig. S2(b) in the supplementary material].

Finally, we compare the single cell analyses to the results from the ensemble fit and find that the ensemble characteristic time is equal or smaller than the mode of the single cell distribution in case of the inlet response [Fig. 3(c) and Fig. S4 in the supplementary material, yellow lines] while the channel traces reveal an opposite trend [Fig. 3(c) and Fig. S4 in the supplementary material, red lines]. These qualitative differences can be understood from the different filtering strategies. While single cell analysis enables evaluation of every single trace, the individual steady-state information is hidden in the intrinsic averaging for the ensemble mean. However, on an ensemble level, Eq. (18) must be fulfilled as well, which is true for all presented conditions assuming $\alpha_{\min}=0.5$ [Figs. 3(a) and 3(b)]. Interestingly, the relationship between single cell analyses, on the one hand, and inlet vs channel response, on the other, seems to be valid also for adherent cells being brought into suspension [Fig. S5 in the supplementary material].

### Measurement bias introduced by single cell analysis

**FIG. 4.**
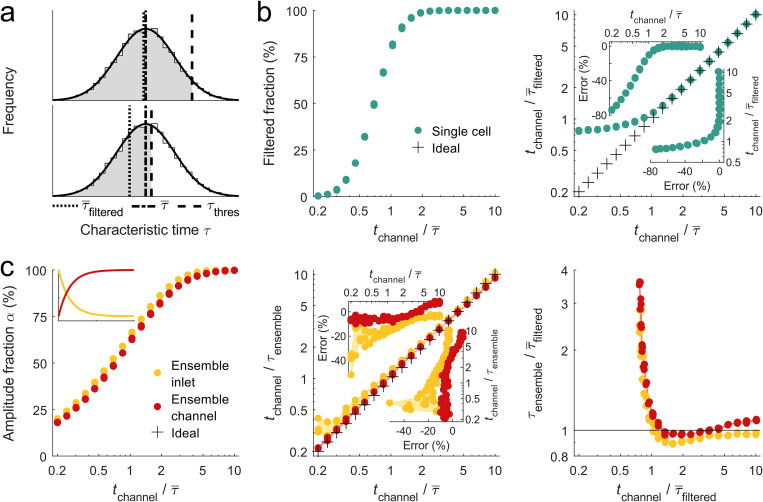
Characteristic time simulation for cell shape dynamics. (a) Lognormal distribution of 10^4^ characteristic times τ with $\bar{\tau }=5\;\mathrm{ms}$ and $\sigma (\log_{10}\tau /\tau_{0})=0.2$ ( $\bar{\tau }$ and σ have been estimated from experimental data, $\tau_{0}$ is set to 1 ms). In experiments, $\bar{\tau }$ is usually not accessible due to finite channel length and is replaced by ${\bar{\tau }}_{\mathrm{filtered}}$, which depends on the upper limit of accessible characteristic times $\tau_{\mathrm{threshold}}$. (b) Filtered fraction of single cell characteristic time distribution as a function of channel translocation time normalized to $\bar{\tau }$ (left panel) and comparison between $t_{\mathrm{channel}}$ normalized to ${\bar{\tau }}_{\mathrm{filtered}}$ and $\bar{\tau }$ (right panel), respectively. Insets show error $({\bar{\tau }}_{\mathrm{filtered}}-\bar{\tau })/\bar{\tau }$ for projections of *x* and *y* axis, respectively. (c) Amplitude fraction as a function of normalized channel translocation time for ensemble analysis of inlet (yellow) and channel data (red). The inset shows exemplary deformation traces for inlet (yellow) and channel (red) shape dynamics. Center panel describes $t_{\mathrm{channel}}$ normalized to ensemble characteristic time as a function $t_{\mathrm{channel}}$ normalized to the median characteristic time from single cell analysis. The right panel compares $\tau_{\mathrm{ensemble}}/{\bar{\tau }}_{\mathrm{filtered}}$ to $t_{\mathrm{channel}}/\tau$ since experimental data do not allow accessing the full characteristic time distribution. Crosses indicate the ideal case without any bias. For every set of parameters, three simulation runs are shown.

We analyzed the impact of different filtering strategies on characteristic times from single cell traces captured inside the channel. As stated above, traces with $\tau_{\mathrm{inlet}/\mathrm{channel}}>\tau_{\mathrm{threshold}}$ are excluded from the analysis. This leads to a shift in the median characteristic time $\bar{\tau }$ toward a lower value ${\bar{\tau }}_{\mathrm{filtered}}$ and introduces a systematic error into single cell analysis. Depending on $\tau_{\mathrm{threshold}}$, the bias can be smaller [Fig. 4(a), top] or bigger [Fig. 4(a), bottom].

We investigated this effect by simulating a characteristic time distribution defined by a lognormal function of median time $\bar{\tau }=5\;\mathrm{ms}$ and a standard deviation of $\sigma (\log_{10}\tau /\tau_{0})=0.2$, with $\tau_{0}=1\;\mathrm{ms}$ [Fig. S6(a) in the supplementary material]. Varying the translocation time $t_{\mathrm{channel}}$ and normalization to $\bar{\tau }$ enables calculation of the filtered fraction of the sample distribution, which corresponds to 50% of all events of the full distribution for $t_{\mathrm{channel}}=0.7\;\bar{\tau }$ and to 99% of all events for $t_{\mathrm{channel}}=2\;\bar{\tau }$ [Fig. 4(b), left panel]. Here, we assumed a minimal amplitude fraction of $\alpha_{\min}=0.5$. A comparison between $t_{\mathrm{channel}}/{\bar{\tau }}_{\mathrm{filtered}}$ and $t_{\mathrm{channel}}/\bar{\tau }$, where $\bar{\tau }$ is experimentally not accessible, reveals an apparent deviation for $t_{\mathrm{channel}}<2\;\bar{\tau }$ [Fig. 4(b), right panel]. Filtering for traces in single cell analysis with translocation times $t_{\mathrm{channel}}$ equal to at least the median relaxation time $\bar{\tau }$ can lead to errors of up to 10%, while we find an error of only 1.5% for $t_{\mathrm{channel}}>2\;\bar{\tau }$ [Fig. 4(b), right panel, top inset].

From these calculations, the measurement bias in single cell analysis can be estimated. An evaluation of the experimentally observed median characteristic times from the eight shape descriptors indicates a range of $1.8\;\mathrm{ms}\leq {\bar{\tau }}_{\mathrm{filtered}}\leq 4.8\;\mathrm{ms}$ for inlet data and $3.5\;\mathrm{ms}\leq {\bar{\tau }}_{\mathrm{filtered}}\leq 7.2\;\mathrm{ms}$ for channel response [Fig. 3(c) and Fig. S4 in the supplementary material]. Normalizing a typical translocation time $t_{\mathrm{channel}}=17\;\mathrm{ms}$ to each median characteristic time yields a ratio from 3.5 to 9.2 for inlet and 2.4 to 4.9 for channel response. This results in an error in single cell analysis between −1.5% and 0.9% [Fig. 4(b), right panel, right inset].

Interestingly, for $t_{\mathrm{channel}}\geq {\bar{\tau }}_{\mathrm{filtered}}$ or $t_{\mathrm{channel}}\geq 0.7\;\bar{\tau }$ , we obtain an error of approximately 20%, which has the same magnitude as the estimated experimental error, e.g., due to pixilation effects of our image acquisition.^38^ Practically, these considerations imply first that the systematic bias due to the inaccessibility of the true median value of the characteristic time is independent of the shape descriptor and can be neglected. Second, our simulations verify that the steady-state of a cell can be extrapolated from translocation times as short (and even shorter) as the characteristic time and that Young's modulus as well as the viscosity can be calculated from such traces.

### Measurement bias introduced by ensemble analysis

Next, we investigated the conditions under which the ensemble average is a good representation of the underlying single cell distribution. Although single cell traces are characterized by an exponential function, the median or mean of many such exponential functions does not necessarily possess exponential behavior. In fact, summation of non-linear functions does not result in a function of the same type without loss of generality.

In a simulation, we addressed this issue and generated characteristic time distributions as described above. Corresponding amplitude values of the traces were taken from a lognormal distribution with center at unity and standard deviation of $\sigma (\log_{10}A)=0.2$ [Fig. S6(b) in the supplementary material]. From each pair of characteristic times and deformation amplitudes, an exponential curve is created, either with a negative [Fig. 4(c), left panel, inset, yellow line] or a positive slope [Fig. 4(c), left panel, inset, red line], representing inlet and channel response, respectively. An ensemble response is calculated from the median over all traces. When calculating the amplitude fraction α of the ensemble response while varying the translocation time $t_{\mathrm{channel}}$ relative to the median characteristic time $\bar{\tau }$ of the single trace distribution, we observe for an increasing translocation time or channel length an increase in α [Fig. 4(c), left panel]. For $t_{\mathrm{channel}}\geq \bar{\tau }$ , we obtain α≥61%, or if we impose $\alpha_{\min}=0.5$, meaning that at least 50% of deformation amplitude are supported by acquired data points within the channel, the translocation time of cells should be chosen to be at least approximately 70% of the expected median relaxation time.

In line with experimental assays, we extract the ensemble relaxation time $\tau_{\mathrm{ensemble}}$ by fitting an exponential function to the median response. Comparing the ensemble relaxation time $t_{\mathrm{channel}}/\tau_{\mathrm{ensemble}}$ to the median of the real single trace characteristic time distribution $t_{\mathrm{channel}}/\bar{\tau }$, both with respect to $t_{\mathrm{channel}}$, we find in almost all conditions a small but obvious underestimation of $\tau_{\mathrm{ensemble}}$ [Fig. 4(c), center panel]. In general, this effect is more pronounced for the inlet response, in particular, for short translocation times. An absolute error below 11% or 9% is observed for the response to inlet or channel stress, respectively, if $t_{\mathrm{channel}}$ is kept between $2\bar{\tau }$ and $10\bar{\tau }$ [Fig. 4(c), center panel, top inset]. The fact of having a finite error even for long translocations times and the apparent differences between inlet and channel computations originates from the non-trivial problem of summation over non-linear functions.

Looking at the experimental data, we find ensemble characteristic times for all eight shape descriptors of $1.5\leq \tau_{\mathrm{ensemble}}\leq 4.4\;\mathrm{ms}$ for the inlet and $3.7\leq \tau_{\mathrm{ensemble}}\leq 8.8\;\mathrm{ms}$ for the channel stress response [Figs. 3(a) and 3(b) and Fig. S2 in the supplementary material]. Normalization of the translocation time to each ensemble characteristic time yields a ratio from 3.9 to 11.3 for inlet and from 1.9 to 4.6 for channel response. This results in an absolute error below 7% for inlet and from −7% to 4% for channel, respectively [Fig. 4(c), center panel, right inset]. A comparison of these values to the systematic error introduced by excluding traces from single cell analysis shows that ensemble averaging results in larger bias but still well below other uncertainties, e.g., pixilation effects.

Since experiments do not permit access to the full characteristic time distribution in single cell analysis nor obtaining the unbiased ensemble characteristic time, we finally compare $\tau_{\mathrm{ensemble}}/{\bar{\tau }}_{\mathrm{filtered}}$ to the normalized translocation time $t_{\mathrm{channel}}/{\bar{\tau }}_{\mathrm{filtered}}$ [Fig. 4(c), right panel]. For $t_{\mathrm{channel}}/{\bar{\tau }}_{\mathrm{filtered}}<1$, i.e., for short translocation times, we find an overestimation of $\tau_{\mathrm{ensemble}}$ compared to single cell analysis as many traces are included in the ensemble characteristic time that do not reach the minimal amplitude fraction. For long translocation times, we find little deviation between the ensemble average and the filtered median of the single cell analysis, as expected. Interestingly, for intermediate translocation times, $1.2\leq t_{\mathrm{channel}}/{\bar{\tau }}_{\mathrm{filtered}}\leq 4$, $\tau_{\mathrm{ensemble}}$ is slightly smaller than ${\bar{\tau }}_{\mathrm{filtered}}$ for both inlet and channel measurements.

### Impact of shape descriptors on effect size after cytoskeletal modifications

**FIG. 5.**
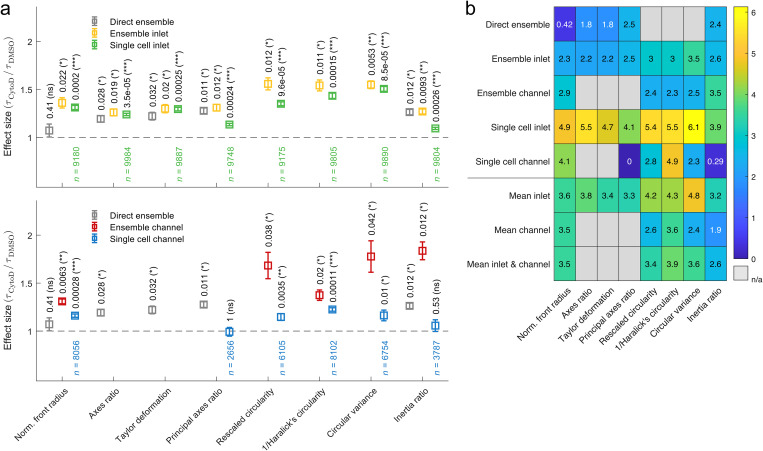
Effect size and score for shape descriptors after cell exposure to CytoD. (a) Effect size for characteristic times of inlet traces (top) and channel traces (bottom) calculated from the ratio of characteristic times of cells treated with 1 *μ*M CytoD and the respective 0.25% (v/v) DMSO vehicle control. Analysis has been performed by fitting an exponential function to the median trace reconstructed from even (yellow) and odd (red) Fourier coefficients (ensemble analysis) as well as by fitting an exponential function to each individual cell trace reconstructed from even (green) and odd (blue) Fourier coefficients (single cell analysis). Since channel relaxation time of principal axes ratio for CytoD treatment exceeds $\tau_{\mathrm{threshold}}$, it is excluded (Fig. S8 in the supplementary material). The gray data points represent a direct ensemble cell analysis without Fourier decomposition. For ensemble analysis, statistical significance has been obtained from a one-parametric *t*-test, while for single cell analysis, statistical significance has been obtained by linear mixed-effect models. The resulting *p*-values are reported on top of data points. Error bars represent standard error of the mean. (b) Heat map: $\mathrm{score}=-\mathrm{effect}\;\mathrm{size}\cdot \log_{10}(\;p\text{-}\mathrm{value})$ to combine effect size and significance level into a scalar. Increasing numbers in color scale represent increasing scores (ns, not significant p≥0.05,p<0.05,∗∗p<0.01,∗∗∗p<0.001). Measurements have been carried out in 1% (v/v) MC-PBS corresponding to a viscosity of 27.8 mPa s at an experimental shear rate of 5105 s^−1^.

Utilizing the results on comparing single cell and ensemble analysis for all eight shape descriptors, we perform measurements on HL60 cells treated with cytochalasin D (CytoD) inhibiting actin polymerization. Cells were incubated with 1 *μ*M CytoD and the corresponding vehicle control [0.25% (v/v) DMSO], respectively (see the Materials and Methods section). Data were acquired by dRT-DC and evaluated as described above (Figs. S7–S10 in the supplementary material). For comparison, also a direct analysis of all time traces, i.e., without Fourier decomposition and reconstruction, was performed [Fig. 5(a) and Fig. S10 in the supplementary material, gray data points]. Here, the cell traces were averaged yielding one ensemble response of deformation expressed by a shape descriptor (Fig. S11 in the supplementary material). The latter approach is computationally less expensive than all other analyses and yields results rapidly but does not allow for a discrimination of inlet and channel effects. Since rescaled circularity, inverse Haralick's circularity, and circular variance reveal a minimum in amplitude inside the channel, data cannot be processed for these shape descriptors without Fourier decomposition.

We define the effect size of cytoskeletal modifications as the ratio between the characteristic times after CytoD treatment and the vehicle control, where $\tau_{\mathrm{CytoD}}/\tau_{\mathrm{DMSO}}=1$ indicates no effect. With the exception of the single cell channel data represented by the principal axes ratio [Fig. 5(a), lower panel, blue data points], all shape descriptors yield an effect size > 1 of varying amplitude and statistical significance. For the direct ensemble analysis, principal axes ratio reveals the largest effect size, while Fourier-based ensemble analysis of the inlet and channel data is best described by rescaled circularity and inertia ratio, respectively. In contrast, single cell data after Fourier reconstruction from the inlet and channel have the largest effect size when being evaluated by circular variance and inverse Haralick's circularity, respectively. In general, we find that shape descriptors calculated from ensemble data reveal larger effect sizes than single cell data for the inlet [Fig. 5(a), upper panel, yellow and green data points] as well as for channel measurements [Fig. 5(a), lower panel, red and blue data points]. It has to be emphasized that this result can be generalized for varying experimental conditions and is, e.g., robust against alterations in the hydrodynamic stress level [Fig. S12 in the supplementary material].

Finally, we combine the results of our effect size analysis and define a score for each shape descriptor,

$$\mathrm{score}=-\mathrm{effect}\;\mathrm{size}\cdot \log_{10}(\;p\text{-}\mathrm{value}),$$ (19)

which takes into account the magnitude of the effect size and the significance level given by the p-value. The score provides a measure, how well a combination of shape descriptor and analysis strategy is suited for probing characteristic times for compound screening. Comparing all approaches presented in this work yields the highest score for single cell analysis at the inlet, a result that could originate from the peak stress at the channel entrance [Fig. 5(b)]. Interestingly, tracking and evaluating individual traces inside the channel also reveal higher scores compared to ensemble averages and emphasizes the importance of single cell analysis to extract quantitative information from compound-response assays. Being computationally less expensive, we also compare inlet and channel characteristic times calculated from ensemble averages. Here, higher scores for traces representing inlet effects were observed.

A detailed look at the different shape descriptors indicates that ensemble analysis at the inlet is best performed using circular variance (score 3.5) and the corresponding channel evaluation should be done by inertia ratio (score 3.5). In contrast, all shape descriptors with the exception of inertia ratio yield high scores for single cell analysis at the inlet (scores between 4.1 and 6.1) while channel traces should be evaluated using normalized front radius (score 4.1) and inverse Haralick's circularity (score 4.9). Ensemble analysis without shape mode decomposition yields the highest scores for principal axes ratio (score 2.5) and inertia ratio (score 2.4) with amplitudes that are equal to or lower than scores using Fourier decomposition and symmetry-based shape reconstruction.

Assessing how well a shape descriptor is suited for the interpretation of cell mechanical measurements independently of the type of analysis, a mean score of analysis strategies was calculated for inlet and channel effects individually and also combined [Fig. 5(b)]. Circular variance (score 4.8) and inverse Haralick's circularity (score 4.3) yield the largest mean score for inlet stress, whereas impact of channel stress is best described by inverse Haralick's circularity (score 3.6) and normalized front radius (score 3.5). The highest overall mean score is reached by inverse Haralick's circularity (score 3.9) and circular variance (score 3.6).

## DISCUSSION

Utilizing dynamic real-time deformability cytometry (dRT-DC), we captured the dynamics of cells passing a microfluidic channel and their deformation in response to two overlapping stress distributions.^17^ The resulting image sequences allow us to extract various time-dependent parameters from dRT-DC data,^23^ which we limited within this work to scalar quantities.^9^ Typical examples of these shape descriptors used in microfluidic methods are axes ratio^3,25,26^ and circularity^15,27^ that represent cellular strain and are thus suitable to determine mechanical properties if the corresponding hydrodynamic stress distribution can be estimated.

While a detailed comparison of shape descriptors for suspended cells in microfluidic systems is elusive, adherent cells have already been characterized in great detail.^8,9^ Here, a link between morphology and cell function was established by shape and motion analysis of motile cells in combination with a standardization of shape parameters. For example, for keratocytes, a close correlation between cell state, shape, and the speed of motion has been found that depends on the self-organization of the membrane and the cytoskeleton. In consequence, cell shape can be predicted by a model based only on cell area, membrane tension, and actin content.^8^ Shape descriptors further allow for quantitative analysis of microscopy images to study cell morphological features in general.^9^

Our study aimed to expand shape analysis to suspended cells inside an extensional as well as in a shear flow. In total, nine different shape descriptors have been analyzed and compared: cell area, front radius, axes ratio, Taylor deformation, principal axes ratio, rescaled circularity, inverse Haralick's circularity, circular variance, and inertia ratio. We utilized Fourier decomposition of cellular shape modes and reconstruction from even and odd coefficients separately to disentangle the superposition of an extensional (inlet) and Poiseuille flow (constriction) inside a microfluidic channel of finite length. In both hydrodynamic geometries, cell response follows an exponential function with characteristic times and amplitudes depending on the respective shape descriptor.

Within this analytical framework, we addressed three questions: First, we investigated the conditions required for cells to reach a steady-state deformation while translocating the microfluidic channel, which is a precondition to determine cell elasticity.^6,34^ The steady-state essentially depends on the viscoelastic property of a cell, i.e., the characteristic time τ that is *a priori* unknown. Since constriction length is usually fixed in microfluidic systems, the only possibility (at least for volumetric flows of constant viscosity) to control the position of steady-state deformation is by translocation time $t_{\mathrm{channel}}$ via changing the flow rate. However, flow rates can only be controlled in a narrow range to ensure sufficiently high shear stress to induce cell deformation and sufficiently small cell velocities to allow dynamic tracking and reduce image blurring. Here, we introduced an amplitude fraction α as a measure of how much of the steady-state amplitude is found inside the constriction. By performing dRT-DC and analyzing the resulting dynamics for different shape descriptors in combination with analytical simulations, we find for α=0.5 the error in τ being below 20%. This implies that steady-state deformation of cells can be predicted from dRT-DC traces for translocation times even shorter than the characteristic time. For $t_{\mathrm{channel}}=2\tau$, the error is only 11% and smaller.

Second, we compared single cell vs ensemble studies, which is relevant to estimate the experimental and computational effort vs data accuracy. For short translocation times, i.e., $t_{\mathrm{channel}}/\bar{\tau }<1$, we find an overestimation of the ensemble characteristic time as traces are included into ${\bar{\tau }}_{\mathrm{ensemble}}$ that do not reach a steady-state. That emphasizes the need for single cell analyses if $\bar{\tau }$ of the sample is unknown. In contrast, for long translocation times, little differences are found between single cell and ensemble studies. When comparing typical translocation times to experimental characteristic times for HL60 cells in this study, we find a ratio of approximately 5 (depending on the shape descriptor), which results in an error of 5% for ensemble and 1.5% for single cell data, which is far below other systematic errors, e.g., due to finite pixel size of our camera system.

Finally, we performed a drug-response assay using cytochalasin D since testing the sensitivity of a method toward cytoskeletal alterations is of essential importance for its application in mechanobiology. Here, we focused on filamentous actin and how the inhibition of its polymerization is reflected by alterations in different shape descriptors. Calculating a score from effect size and statistical significance, we demonstrated that single cell analysis at the inlet provides highest sensitivity almost independent of how deformation is quantified. In contrast, when measurements are limited to the constant flow profile inside the channel to compute elasticity and viscosity, inverse Haralick's circularity provides the highest score. This capability to provide a quantitative decision criterion for the selection of a shape descriptor in dependence of the hydrodynamic geometry might be of essential importance in any project where mechanical properties are being used to study the response of suspended cells to unknown compounds.

## CONCLUSION

In summary, we have demonstrated the potential of different shape descriptors for quantifying the dynamics of a cell passing a microfluidic constriction. Analysis has been performed under different conditions in response to a drug inhibiting actin polymerization. We performed the data analysis on three different levels of depth, spanning a range from directly calculating the shape descriptors to performing Fourier analysis to disentangle inlet and channel contributions before investigating ensemble and single cell response, separately. Importantly, our studies demonstrate that a steady-state deformation of cells is not required to calculate viscoelastic properties and to compare the stress response of cells under different conditions, e.g., drug treatment, as along as a sufficient part of the shape dynamics inside a microfluidic channel is known and can be quantified.

Our investigations include not only an in-depth comparison of a variety of shape descriptors for viscoelastic cell characterization in microfluidic systems but also provide experimentalists with a framework to estimate systematic errors introduced by data analysis strategy and translocation time, i.e., acquisition time per cell.

## SUPPLEMENTARY MATERIAL

See the supplementary material for supporting information and equations.

## Data Availability

The data that support the findings of this study are available from the corresponding author upon reasonable request.
